# Bilateral Congenital Agenesis of the Long Head of the Biceps Tendon: The Beginning

**DOI:** 10.1155/2016/4309213

**Published:** 2016-01-21

**Authors:** Francisco Rego Costa, Cátia Esteves, Lina Melão

**Affiliations:** Department of Radiology, São João Hospital, Alameda Professor Hernâni Monteiro, 4200-319 Porto, Portugal

## Abstract

The biceps brachii muscle is prone to variants but absence of the long head of the biceps (LHB) tendon is an exceptionally rare anomaly. This report concerns the fourth case of bilateral congenital absence of the LHB tendon and presents the ultrasonography (US) and magnetic resonance (MR) findings. Our case has the peculiarity of being the first in which bilateral LHB tendon agenesis is not associated with rotator cuff or labral tears.

## 1. Introduction

Absence of the long head of the biceps (LHB) tendon is a very unusual anomaly. We report the fourth case of bilateral congenital absence of the LHB tendon in the English literature. Ultrasonography (US) and magnetic resonance (MR) findings are exposed.

## 2. Case Report

A 29-year-old male, Muay Thai amateur fighter, presented with right anterior shoulder pain at rest that exacerbated with overhead activities. The pain was moderate for months but worsened in the last few weeks, specially after sports activity. He had no symptoms in the left shoulder.

Although the patient is a sportsman, there was no history of a significant lesion/traumatic event involving the upper limbs, including shoulder dislocation. He also denied orthopedic surgery to both shoulders.

The physical exam was almost unremarkable. The uppercut and Speed's tests were negative and the “Popeye” sign was absent. Digital pressure over the bicipital groove did not elicit pain. Although the rotatory stress test and the lateral decubitus load and shift test were negative, the anterior apprehension sign was doubtful. Also there was some limited range of motion when the patient tried to elevate the left arm with the forearm in supination. Overall, there was no clear evidence of biceps pathology or anterior instability.

**Figure 1:**
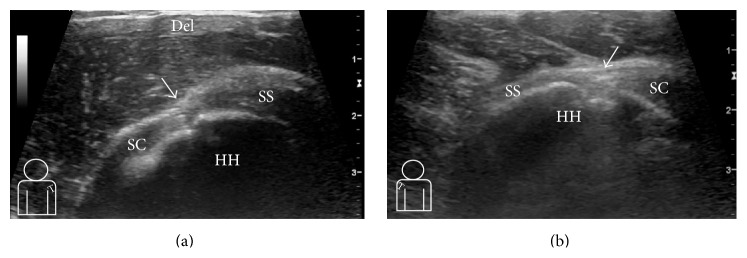
Ultrasound (US) images of both shoulders. (a) US parasagittal image of the left shoulder rotator interval. The long head of the biceps tendon is not identifiable in its normal position (white arrow). (b) US parasagittal image of the right shoulder rotator interval. The long head of the biceps tendon is not discernible in its normal position (white arrow). HH = humeral head; SS = supraspinatus tendon; SC = subscapularis tendon; Del = deltoid muscle.

On the ultrasonography of the painful and contralateral shoulder we were not able to demonstrate the LHB tendon in its groove (Figure 1), suggesting the presence of a tear or agenesis. There was no evidence of rotator cuff tears, bursitis, or effusion.

**Figure 2:**
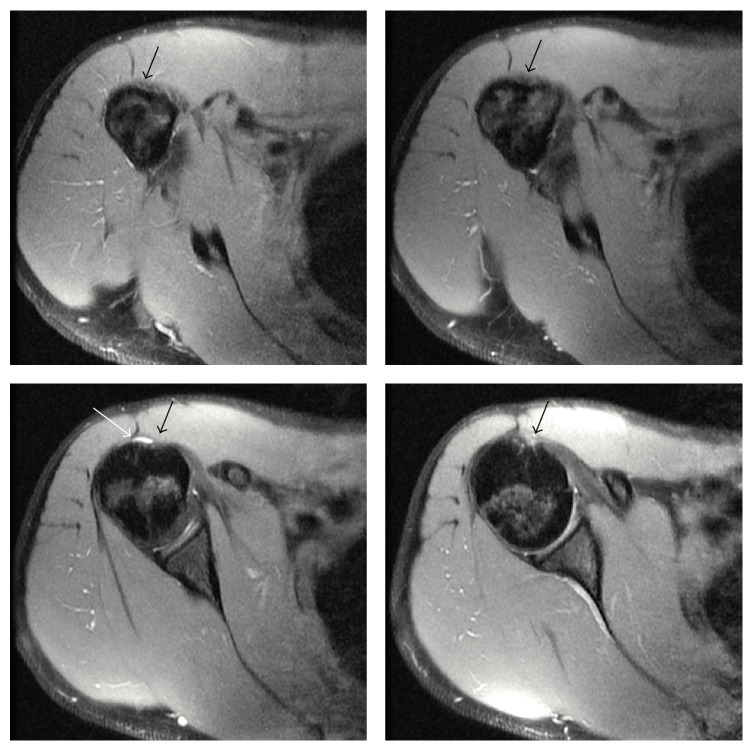
Axial DP FS SE MR sequential images of the right shoulder (TR = 2540; TE = 11.18; NEX = 2; EC = 1) demonstrating a shallow bicipital groove (black arrows) and absence of the long head of the biceps tendon. There is a small amount of fluid in the subacromial/subdeltoid bursa (white arrow), without pathologic significance.

**Figure 3:**
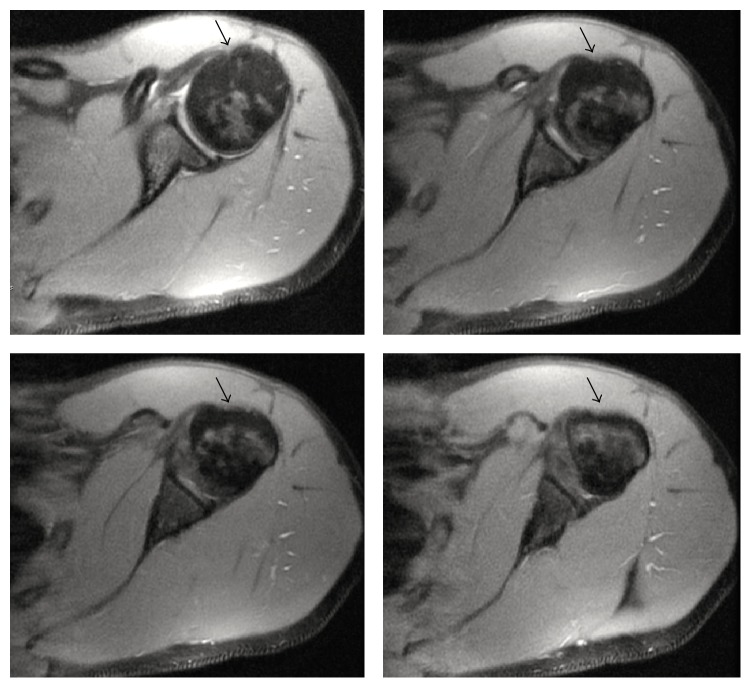
Axial DP FS SE MR sequential images of the left shoulder (TR = 2540; TE = 11.18; NEX = 2; EC = 1) showing a shallow bicipital groove (black arrows) and absence of the long head of the biceps tendon.

**Figure 4:**
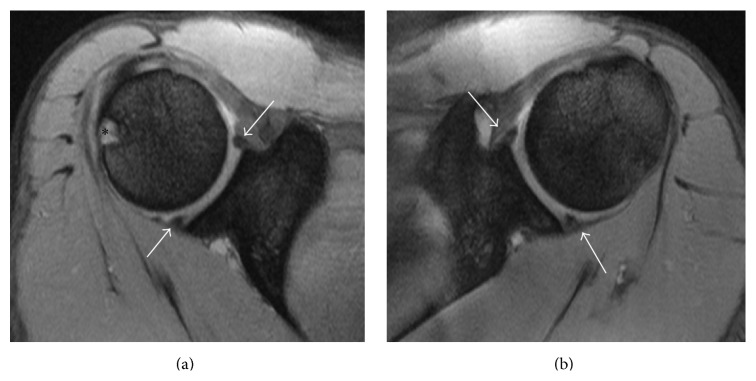
Axial DP FS SE MR images of the right (a) and left (b) shoulder (TR = 475; TE = 13.43; NEX = 1; EC = 1) showing normal labrum morphology bilaterally, without evidence of tears (white arrows). In (a) there is a cyst beneath the lateral humeral head margin, without pathologic significance (asterisk).

**Figure 5:**
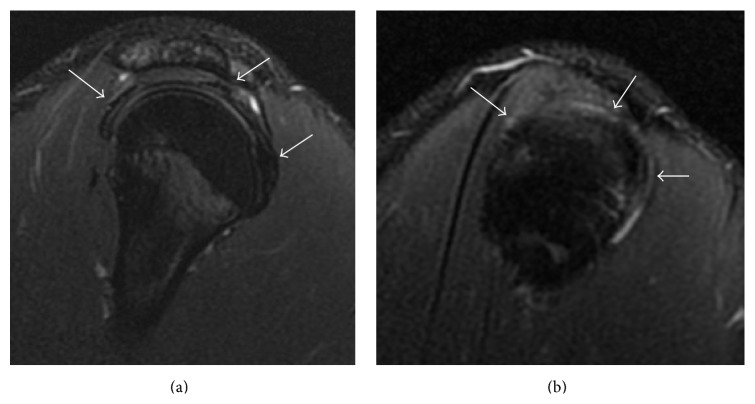
Sagittal T2 FS SE MR images of the right shoulder (TR = 2600; TE = 63.9; NEX = 3; EC = 1) (a) and (b) demonstrating the rotator cuff tendons (white arrows) with normal thickness and hypointensity, without tears.

**Figure 6:**
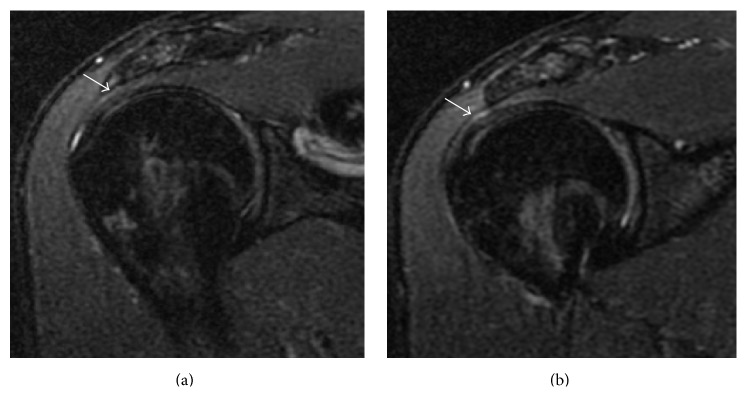
Coronal T2 FS SE MR images of the right shoulder (TR = 3500; TE = 63.2; NEX = 2; EC = 1) (a) and (b) showing the normal supraspinatus tendon long axis (white arrows), without tears.

**Figure 7:**
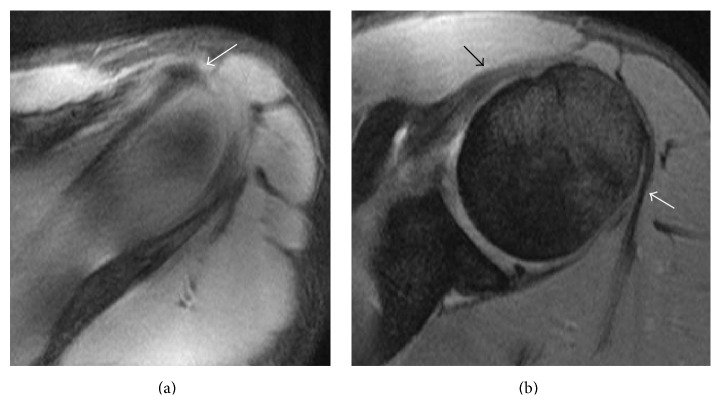
Axial DP FS SE MR images of the left shoulder (TR = 475; TE = 13.4; NEX = 2; EC = 1). (a) The supraspinatus tendon is visible, without any discontinuity (white arrow). (b) The subscapularis tendon (black arrow) and the infraspinatus tendon (white arrow) show normal thickness and hypointensity, without tears.

Magnetic resonance imaging of both shoulders revealed bilateral absence of the LHB tendon and shallow intertubercular sulci (Figures 2 and 3). There was no evidence of rotator cuff tears on the right side or labral tears bilaterally (Figures 4, 5, and 6). As the patient had no symptoms on the left side, only axial plane MR images were obtained, with the main purpose of proving bilateral agenesis. On these images there was no evidence of rotator cuff abnormalities (Figure 7).

## 3. Discussion

The biceps brachii muscle is one of the most diverse in the human body; however, congenital absence of the long head of the biceps tendon is a rare anomaly [1–7]. Frequently there are associated findings such as hypoplastic intertubercular groove [1–3] and shoulder instability [4–7]. Unilateral absence is associated with other skeletal and nonskeletal congenital anomalies in 57% of cases [1, 4–6], including at least one case associated with VATER complex (vertebral defects, anal atresia, tracheoesophageal fistula with esophageal atresia, and radial and renal anomalies) [4]. Bilateral absence is extremely uncommon with only three prior reports in the English literature [1–3]. Solely one of these three cases presented with an additional skeletal abnormality (radial ray) [1].

In our case, the patient was healthy and physically active and no other congenital anomalies were evident. As in other reported cases, bilateral agenesis of the LHB tendon is most commonly diagnosed in a young adult patient complaining of nonspecific shoulder pain [1–3]. Evidence of shallow intertubercular groove is the “rule,” and our case is not an exception. The finding of a shallow groove is very helpful in the differential diagnosis, favoring agenesis over the much more common diagnosis of a biceps tear.

Our case is the first to describe bilateral absence of the LHB tendon not associated with rotator cuff or labral tears. No other significant abnormality was discernible in both shoulders. These findings support that agenesis of the LHB tendon can, by itself, be a source of symptoms and functional deficit. Based on other cases described, which emphasized the role of the LHB tendon as a shoulder stabilizer [3, 8, 9], we believe that this case can represent the earliest stage of the shoulder disease process generated by the absence of the LHB tendon.
